# Combination therapy for platinum-resistant ovarian cancer: a novel at-home regimen with envafolimab, lenvatinib, and etoposide

**DOI:** 10.1093/oncolo/oyaf210

**Published:** 2025-07-14

**Authors:** Bo Ding, Tianxiang Yu, Shiya Zheng, Jingyun Xu, Feng Ji, Hao Lin, Xiang Zhao, Shanhu Qiu, Yang Shen

**Affiliations:** Department of Obstetrics and Gynecology, Zhongda Hospital, School of Medicine, Southeast University, Nanjing 210009, China; Nanjing Key Laboratory of Intelligent Obstetrics and Gynecology Medicine, Zhongda Hospital, School of Medicine, Southeast University, Nanjing 210009, China; Department of Obstetrics and Gynecology, Zhongda Hospital, School of Medicine, Southeast University, Nanjing 210009, China; Department of Oncology, Zhongda Hospital, School of Medicine, Southeast University, Nanjing 210009, China; Department of Obstetrics and Gynecology, Zhongda Hospital, School of Medicine, Southeast University, Nanjing 210009, China; Nanjing Key Laboratory of Intelligent Obstetrics and Gynecology Medicine, Zhongda Hospital, School of Medicine, Southeast University, Nanjing 210009, China; Nanjing Key Laboratory of Intelligent Obstetrics and Gynecology Medicine, Zhongda Hospital, School of Medicine, Southeast University, Nanjing 210009, China; Department of Clinical Science and Research, Zhongda Hospital, School of Medicine, Southeast University, Nanjing 210009, China; Nanjing Key Laboratory of Intelligent Obstetrics and Gynecology Medicine, Zhongda Hospital, School of Medicine, Southeast University, Nanjing 210009, China; Department of Clinical Science and Research, Zhongda Hospital, School of Medicine, Southeast University, Nanjing 210009, China; State Key Laboratory of Neurology and Oncology Drug Development, Jiangsu Simcere Diagnostics Co., Ltd, Nanjing Simcere Medical Laboratory Science Co., Ltd, Nanjing 210042, China; Department of General Practice, Zhongda Hospital, Institute of Diabetes, School of Medicine, Southeast University, Nanjing 210009, China; Department of Obstetrics and Gynecology, Zhongda Hospital, School of Medicine, Southeast University, Nanjing 210009, China; Nanjing Key Laboratory of Intelligent Obstetrics and Gynecology Medicine, Zhongda Hospital, School of Medicine, Southeast University, Nanjing 210009, China

**Keywords:** ovarian cancer, platinum-resistant, at-home regimen, combination therapy

## Abstract

**Background:**

Effective and tolerable treatment for patients with platinum-resistant recurrent ovarian cancer remains a great challenge in clinical practice. This study aimed to evaluate the efficacy and safety of envafolimab, the first subcutaneously administered programmed death ligand 1 (PD-L1) inhibitor, combined with lenvatinib and etoposide in patients with platinum-resistant recurrent ovarian cancer.

**Methods:**

This was an open-label, single-arm, phase 2 trial (ENLEN-OC-001). Patients with platinum-resistant recurrent ovarian cancer were eligible for inclusion who were administered envafolimab subcutaneously on day 1 and lenvatinib and etoposide orally on days 1-14, with 21 days as a cycle. After 6-10 cycles, envafolimab and lenvatinib were taken as maintenance therapy until intolerable toxicity, disease progression, withdrawal of consent, or finishing 24 months of treatment. The primary endpoint was objective response rate (ORR), and the secondary endpoints included disease control rate (DCR), progression-free survival (PFS), overall survival (OS), and safety.

**Results:**

Of the screened 28 patients, 21 were included, with 18 being assessed for the efficacy and safety. The ORR was 44.4% (95% CI 21.5%-69.2%), and the DCR was 83.3% (95% CI 58.6%-96.4%). The median PFS was 10.2 months (95% CI 5.6-not applicable [NA]), and the median OS was 21.3 months (95% CI 6.8-NA). The most common grade 3/4 adverse events were leukopenia (27.8%) and thrombocytopenia (16.7%). No serious adverse events or treatment-related deaths were reported. No changes were observed in the depression, anxiety, and quality of life following treatment.

**Conclusion:**

Envafolimab combined with lenvatinib and etoposide showed promising efficacy and tolerable safety for patients with platinum-resistant recurrent ovarian cancer.

**ClinicalTrials.gov identifier:**

NCT05422183

Lessons learned The 3-agent combination therapy with envafolimab, lenvatinib, and etoposide showed favorable therapeutic effects and manageable toxicity in patients with platinum-resistant recurrent ovarian cancer and represented a potential treatment option for at-home care.

**Trial information**

**Table:** 

Trial information	
**Disease**	Platinum-resistant ovarian cancer
**Stage of disease/treatment**	I-IV
**Prior therapy**	No designated number of regimens
**Type of study**	Phase II
**Primary endpoints**	Objective response rate
**Secondary endpoints**	Disease control rate, progression-free survival, overall survival, and safety

## Additional details of endpoints or study design

### Study design and patients

This study was an open-label, single-arm, phase 2 trial (ClinicalTrials.gov registration no. NCT05422183), which was conducted from June 2022. The study protocol was approved by the Institutional Review Board at Zhongda Hospital, Southeast University (approval no. 2022ZDSYLL184-P01). Patients were included if they met the following criteria: (1) aged 18-75 years; (2) a histological diagnosis of recurrent ovarian cancer; (3) platinum-resistant (defined as progression within 6 months after the last platinum treatment) or platinum-refractory (defined as progression during the initial platinum-based treatment); (4) at least one measurable lesion as defined by Response Evaluation Criteria in Solid Tumors version (RECIST) 1.1 (≥10 mm for non-nodal lesions and ≥15 mm along the short axis for nodal lesions); (5) an Eastern Cooperative Oncology Group performance status (ECOG-PS) of 0-2; (6) a life expectancy of >12 weeks; and (7) adequate bone marrow function (neutrophil count ≥1500 cells/μL, platelet count ≥100 000 cells/μL, and hemoglobin concentration of ≥9.0 g/dL), liver function (total bilirubin ≤1.5 times the upper limit of normal, aspartate transaminase or alanine transaminase ≤2.5 times the upper limit of normal), and renal function (serum creatinine ≤1.5 times the upper limit of normal or creatinine clearance rate of ≥60 mL/min calculated using the Cockcroft–Gault formula).

Patients were excluded if they had (1) a previous or current diagnosis of another malignant tumor(s) (excluding treated skin basal cell carcinoma and cervical carcinoma in situ); (2) a prior use of PD-1/PD-L1 inhibitors; (3) an allergy to large-molecule protein agents or drug components; or (4) difficulty in swallowing, and so on.

### Treatment regimens

Patients underwent a 21-day cycle of the combined therapy, which included a subcutaneous injection of envafolimab (400 mg) on day 1, a daily oral administration of lenvatinib (12 mg/day for patients weighing ≥60 kg and 8 mg/day for patients weighing <60 kg), and an oral administration of etoposide (50 mg once daily) on days 1-14. After 6-10 cycles, the patients continued taking envafolimab and lenvatinib as maintenance therapy until intolerable toxicity, disease progression, withdrawal of consent, or finishing 24 months of treatment.

The investigators adjusted the dosage based on drug-related toxicities and anticipated clinical benefits, such as temporary discontinuation or dose reduction. Discontinuation of the drug use lasted for no longer than 2 weeks. Two dosage reductions were permitted: initially adjusting lenvatinib to 8 mg/day for patients weighing ≥60 kg or 4 mg/day for patients weighing <60 kg, followed by a further reduction to 4 mg daily or every other day. For etoposide, an initial reduction to 50 mg on days 1-12 and then on days 1-10 in the subsequent cycle was allowed. However, there were no dosage adjustments for envafolimab.

Depression, anxiety, and quality of life (QoL) were assessed by the self-rating depression scale (SDS), self-rating anxiety scale (SAS), and functional assessment of cancer therapy–ovarian cancer (FACT-O), respectively, before and after the completion of 2 treatment cycles. Lower scores for the SDS and SAS were indicative of better psychological well-being, while higher scores of FACT-O were of better QoL.

The investigators evaluated the lesions and tumor response using computerized tomography scans or magnetic resonance imaging according to the RECIST v.1.1 criteria initially as a baseline, every 2 to 3 cycles during the combined therapy, and every 3 to 3 months during the maintenance therapy. Confirmation of a complete (CR) or partial (PR) response was required at least 4 weeks after the initial response. Disease progression was documented 4 to 6 weeks after progression. Discontinuation of study treatment due to progression was monitored every 2 to 3 months until the subsequent cancer therapy or the end of the study, whichever occurred first. Adverse events were graded based on the National Cancer Institute Common Terminology Criteria for Adverse Events (NCI-CTCAE; version 5.0) from treatment initiation to 90 days post-last cycle, with assessments every 2 weeks during the follow-up period.

Formalin-fixed, paraffin-embedded tumor tissues obtained from the initial surgery were collected initially at baseline. Immunohistochemistry was performed to assess PD-L1 expression in the tissue samples using an anti-human PD-L1 rabbit monoclonal antibody (clone: SP263) on the Ventana Benchmark Ultra platform. The PD-L1 results were interpreted based on the combined positive score (CPS) criteria, with the CPS calculated as a percentage of PD-L1–stained tumor cells and tumor-associated immune cells relative to the total number of tumor cells.

### Outcomes

The primary endpoint in this trial was the ORR, defined as the percentage of patients exhibiting either CR or PR based on the RECIST v.1.1 criteria. The secondary endpoints included OS, PFS, DCR, and the frequency and severity of adverse events. OS was defined as the duration from treatment initiation to death from any cause. PFS was defined as the period from treatment initiation to disease progression or death (whichever occurred first) or the last assessment of PFS for surviving patients without progression. DCR was defined as the proportion of patients with CR, PR, or stable disease (SD).

### Statistical analysis

The sample size was performed using 1-sided exact text by PASS 2021. The trial was designed with 80% statistical power and a 1-sided type I error rate of 5%. Based on an estimated ORR of 40% in this study versus the reference ORR of 15% from previous studies (KEYNOTE-100,^1^ JAVELIN,^2^ and NINJA^3^), a minimum of 18 participants was required to detect the significance. Considering a dropout rate of 10%, the required sample size was adjusted to 20 patients.

Descriptive statistics were used to summarize demographic characteristics and safety data. Continuous variables are described using the median and interquartile range (IQR), while categorical variables are described using the frequency and percentage. Survival outcomes were analyzed using the Kaplan–Meier method and compared with the log-rank test. In addition, 95% CIs were computed using the Clopper–Pearson method. The statistical analyses in this research were conducted using the R statistical software (version 4.3.3).

**Drug information**

**Table:** 

Drug information	
**Generic/working name**	Envafolimab
**Company name**	KN035
**Drug type**	Antibody
**Drug class**	Immunetherapy
**Dose**	200 mg
**Route**	Subcutaneous
**Schedule of administration** Patients received envafolimab as a single fixed dose of 400 mg on day1 in each 21-day cycle	
**Generic/working name**	Lenvatinib
**Company name**	Lenvima
**Drug type**	Small molecule
**Drug class**	Tyrosine kinase inhibitor
**Dose**	4 mg
**Route**	Oral
**Schedule of administration** Patients received lenvatinib daily (12 mg/day for patients weighing ≥60 kg and 8 mg/day for patients weighing <60 kg)	
**Generic/working name**	Etoposide
**Company name**	Laspetot
**Drug type**	Chemotherapy
**Drug class**	Chemotherapy
**Dose**	50 mg
**Route**	Oral
**Schedule of administration** Patients received etoposide as a single fixed dose of 50 mg on day 1-14 in each 21-day cycle	

**Patient characteristics**

**Table:** 

Patient characteristics	
Number of patients	18
Stage	I: *n* = 2; II: *n* = 2; III: *n* = 10; IV: *n* = 4
Number of prior systemic therapies: median (range)	54.5 (41-75)
Performance status: ECOG 0 or 1	16
Performance status: ECOG 2 or above	2
Cancer types or histologic subtypes	High-grade serous carcinoma, 15; clear cell carcinoma, 3

**Primary assessment method**

**Table:** 

Primary assessment method	
**Title**	Objective response rate
**Number of patients screened**	28
**Number of patients enrolled**	21
**Number of patients evaluable for toxicity**	18
**Number of patients evaluated for efficacy**	18
**Evaluation method**	RECIST 1.1

## Outcome notes

### Baseline characteristics of patients

Twenty-eight patients underwent screening from June 18, 2022, to March 31, 2025, with 21 eligible for this study (Figure S1). Seven patients were excluded because 5 had non-assessable target lesions, 1 underwent ileus surgery, and 1 had a projected lifespan of ≤3 months. The remaining 21 patients received at least 1 treatment cycle. Because of the lack of post-baseline tumor assessment in 3 patients who discontinued their medication after receiving 2 cycles of free treatment, the therapeutic outcomes of 18 patients were finally assessed. At the data cutoff point, 4 (22.2%) patients remained on treatment.

Their median age was 54.5 years (IQR: 41-75), and BMI was 24.4 kg/m^2^ (IQR: 12-37) (Table S1). Three patients (16.7%) were diagnosed with clear cell carcinoma, and 15 patients (83.3%) were diagnosed with high-grade serous carcinoma. Their median number of prior chemotherapies was 3, and 7 patients (38.9%) had received more than 3 lines of therapy. Among the 18 assessable patients, 8 (44.4%) had tumor samples suitable for PD-L1 staining. Five patients (27.8%) had a CPS of ≥1, and 3 patients (16.7%) had a CPS of ≥10.

### Survival outcomes

**Figure 1.**
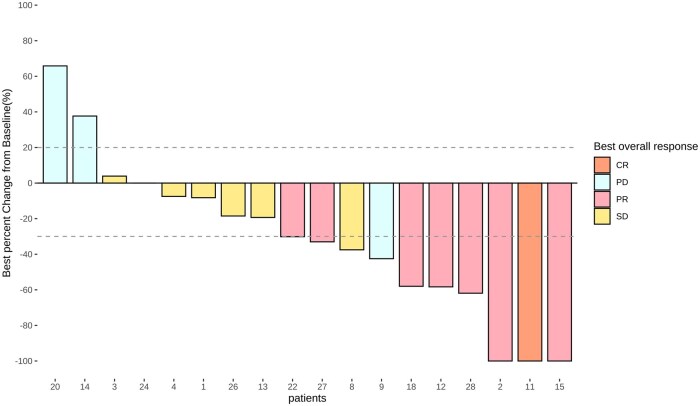
Best changes percentage change from baseline in the target lesion.

**Figure 2.**
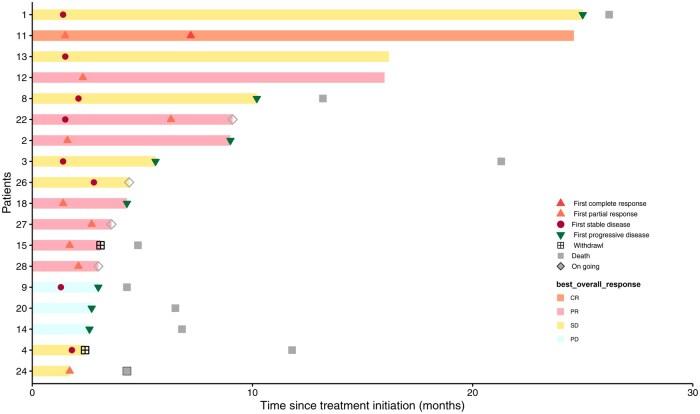
Treatment exposure and response duration.

**Figure 3.**
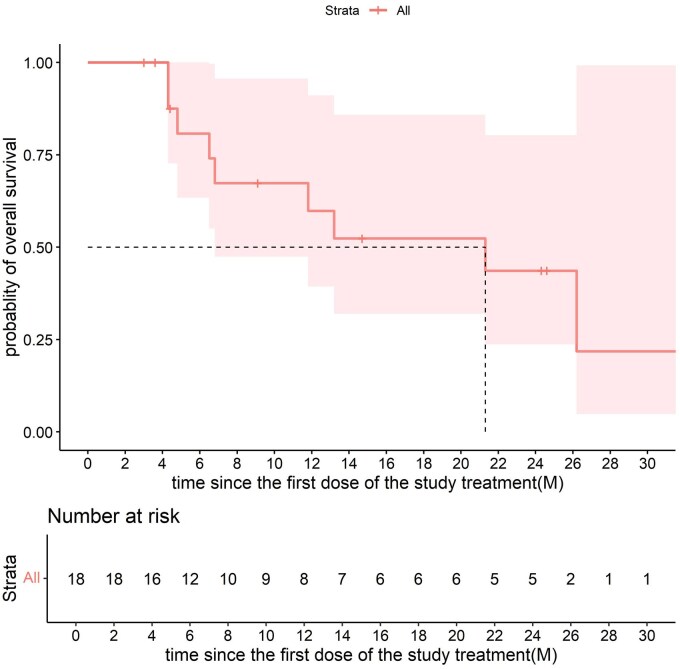
The Kaplan–Meier curves of OS.

**Figure 4.**
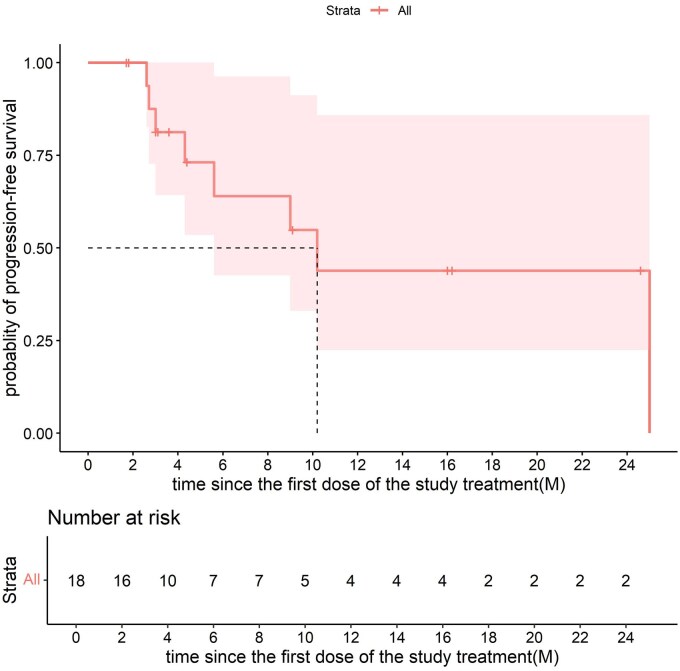
The Kaplan–Meier curves of PFS.

Among the 18 patients, 8 (44.4%) showed objective responses, and 15 (83.3%) had objective control. Seven patients (38.9%) showed a maintained disease control for at least 6 months (Figures 1 and 2; Table 1). The median OS was 21.3 months (95% CI 6.8-NA) (Figure 3, Table 1), and the median PFS was 10.2 months (95% CI 5.6-NA); Figure 4, Table 1). The previous testing, therapies, and survival data of the 18 patients are summarized in Table S2.

**Table 1.** Summary of the clinical response and survival outcomes.

Variables	All patients (*n* = 18)
**Overall response, *n* (%)**	
** Complete response**	1 (5.6)
** Partial response**	7 (38.9)
** Stable disease**	7 (38.9)
** Progressive disease**	3 (16.7)
**ORR, % (95% CI)**	44.4 (21.5-69.2)
**DCR, % (95% CI)**	83.3 (58.6-96.4)
**PFS, months, median (95%CI)**	10.2 (5.6-NA)
**OS, months, median (95% CI)**	21.3 (6.8-NA)

The responses were assessed with RECIST v.1.1. Abbreviations: DCR, disease control rate; ORR, objective response rate; OS, overall survival; PFS, progression-free survival; RECIST, Response Evaluation Criteria in Solid Tumors.

### Safety analysis

Doses were reduced for 10 patients (55.6%) who received etoposide, with 7 having one dose reduction and 3 having 2 dose reductions. The dose adjustments for etoposide primarily occurred during or after the first or second treatment cycle. Additionally, the lenvatinib dose was reduced for 3 patients (16.7%) due to grade 3 thrombocytopenia.

The 18 patients experienced different degrees of treatment-related adverse events (Table 2). The most common adverse events included leucopenia (77.8%) and thrombocytopenia (50%). Hypothyroidism was the predominant immune-related adverse event, affecting 4 patients (22.2%). The most frequent grade 3-4 adverse events were leucopenia (27.8%, 5/18) and thrombocytopenia (16.7%, 3/18). No serious adverse events or treatment-related deaths were reported.

**Table 2.** Treatment-related adverse events in the safety population, *n* (%).

Adverse events	Grade 1-2	Grade ≥3	Any grade
**Hematological**			
** Leukopenia**	9	5 (27.8)	14 (77.8)
** Thrombocytopenia**	6 (33.3)	3 (16.7)	9 (50)
** Anemia**	6 (33.3)	1 (5.5)	7 (38.9)
**Non-hematological**			
** Fatigue**	7	0	7 (38.9)
** Anorexia**	5 (27.8)	0	5 (27.8)
** Diarrhea**	2 (11.1)	0	2 (11.1)
** Vomiting**	2 (11.1)	0	2 (11.1)
** Nausea**	6 (33.3)	0	6 (33.3)
** Hypothyroidism**	4 (22.2)	0	3 (16.7)
** Hyperuricemia**	2 (11.1)	0	2 (11.1)
** Hand-foot syndrome**	2 (11.1)	0	2 (11.1)
** Mucositis**	3 (16.7)	2 (11.1)	5 (27.8)
** Hypokalemia**	3 (16.7)	1 (5.5)	4 (22.2)
** Urinary infections**	3 (16.7)	0	3 (16.7)
** Ileus**	2 (11.1)	1 (5.5)	3 (16.7)
** Ascites**	2 (11.1)	2 (11.1)	4 (22.2)
** Urinary obstruction**	0	1 (5.5)	1 (5.5)
** Hypertension**	2 (11.1)	1 (5.5)	3 (16.7)

### Variations in psychological status and QoL before and after treatment

The SDS and SAS scores did not differ significantly before and after the 2-cycle treatment (*P* > .05; Table S3). Similarly, the FACT-O scores were also not different before and after treatment (*P* > .05).

## Discussion

### Main findings

Our study, which is, to our knowledge, the first trial that assessed the efficacy and safety of envafolimab in combination with lenvatinib and etoposide for platinum-resistant recurrent ovarian cancer. Our results showed that this 3-agent combined therapy exhibited significant antitumor effects in patients with platinum-resistant recurrent ovarian cancer, with manageable toxicity and favorable outcomes on mental status and QoL. In our study, envafolimab was administered via subcutaneous injection, and lenvatinib and etoposide were administered orally, offering a more convenient and comfortable treatment regimen for at-home care.

### Interpretation

ICIs in combination with the use of anti-angiogenic therapy and chemotherapy have demonstrated improved outcomes for patients with platinum-resistant recurrent ovarian cancer.^4^ Current treatment options for platinum-resistant recurrent ovarian cancer are limited, but there is growing interest in combination therapies involving programmed death 1 (PD-1)/PD-L1 inhibitors and other medications. A recent study by Xia et al. indicated that the combination of camrelizumab and famitinib produced an ORR of 24.3% in patients with advanced ovarian cancer.^5^ A phase 1 b trial involving anlotinib and TQB2450 in 34 patients with platinum resistance demonstrated an ORR of 47.1%, a median PFS of 7.8 months, and a durable response lasting at least 8 months in 61.3%.^6^ Our study was the first one to use envafolimab, the first subcutaneous single-domain anti–PD-L1 antibody,^7^ to treat platinum-resistant recurrent ovarian cancer in combination with lenvatinib and etoposide, showing an ORR of 44.4%, a DCR of 83.3%, and a median PFS of 10.2 months, indicating that the “home-stay” regimen had effective antitumor activity against platinum-resistant recurrent ovarian cancer.

The AEROC study reported an ORR of 54% and 8.1 months PFS in 35 patients who were treated with apatinib and oral etoposide.^8^ Partly inconsistent with that outcome, our study observed an ORR of 44.4%, albeit with a longer PFS of 10.2 months. In our study, 3 patients who did not receive prior use of PARP inhibitors, bevacizumab, or anti-vascular TKIs showed better outcomes significantly (Table S2). It seems likely that patients presenting with baseline relapse following multi-line chemotherapeutic regimens subsequent to the clinical adoption of PARP inhibitors and bevacizumab in routine practice may demonstrate compromised therapeutic efficacy when undergoing triple-agent combination regimens.

Our study observed favorable outcomes in 3 patients diagnosed with clear cell carcinoma, whose PFS was over 8 months during the study period, and 2 PR and 1 SD were observed. Zamarin et al. found that patients with ovarian clear cell carcinoma (OCCC) had a response rate to immunotherapy approximately 5-fold higher than those with other pathological subtypes.^9^ The study by Peng et al. provided compelling evidence that combining immunotherapy with anti-angiogenic therapy enhanced clinical benefit in patients with OCCC.^10^ Upregulation of HNF1 homeobox B (HNF1B/HNF-1β) in OCCC cells can induce the expression of nuclear factor kappa B (NF-κB), which then induces the expression of PD-1 and its ligand PD-L1, facilitating the immune escape of tumor cells.^11^ Furthermore, mutations in the AT-rich interaction domain 1A (ARID1A) gene have been associated with microsatellite instability and increased tumor mutation load.^12^ These factors ­promote the recognition of neoantigens by dendritic cells and T lymphocytes, resulting in an immune response against cancer cells. Accordingly, OCCC has a unique immune microenvironment, making immunotherapy a potentially effective therapeutic strategy for clear cell carcinoma compared to other pathological types.^13^

In our study, 8 patients were tested for PD-L1 expression. We did not find a significant association between antitumor activity and PD-L1 expression. The value of PD-L1 expression as a predictor of immunotherapy in ovarian cancer remains controversial. In a trial involving pembrolizumab and bevacizumab, patients with a PD-L1 tumor proportion score of ≥1% had a higher ORR (52.6%), suggesting that patients with high PD-L1 expression responded better to treatment.^14^ However, the outcome from a trial with nivolumab and bevacizumab indicated that the PD-L1-negative population had a higher response rate.^15^ Our study is similar to the results of the JAVELIN solid tumor trial, showing that PD-L1 expression assays may not be able to fully predict patient response to immune-based chemotherapy, possibly due to the complex immune microenvironment of ovarian cancer itself.^2^ Therefore, further research is needed to identify more reliable biological markers for PD-1/PD-L1 inhibitor therapy.^16^

One patient in our study demonstrated a positive homologous recombination deficiency (HRD) status with a score of 89 and showed a CR. This implies that positive HRD expression may indicate a favorable response to the therapy among patients with platinum-resistant recurrent ovarian cancer. While HRD has been used to identify patients with ovarian cancer who may benefit clinically from PARP inhibitors, a gap remains regarding the relationship between HRD and immunotherapy efficacy in ovarian cancer. In other cancers, such as non–smooth-cell lung cancer and pancreatic cancer, HRD has shown potential as a biological predictor of the efficacy of immunotherapy.^17^^,^^18^ Importantly, immunotherapy can also be advantageous for patients without HRD positivity. The DUO-O trial, which assessed the use of durvalumab in combination with platinum-based chemotherapy and bevacizumab in patients with advanced ovarian cancer, showed that immunotherapy combined with chemotherapy and targeted therapy benefited patients irrespective of their BRCA1/BRCA2 DNA repair-associated (BRCA1/2) mutational status.^19^

Our study showed a manageable toxicity, and no new adverse events emerged compared to previous studies. In our study, 7 patients experienced grade 3-4 adverse events, with leukopenia and thrombocytopenia being the most common. Etoposide was identified as the primary contributor to adverse events in our study. The discomforts reported by most patients were relieved within a week of intermittent etoposide administration during the trial, and the symptoms of patients experiencing adverse events improved following adjustments to the etoposide dosage. These findings were consistent with the safety data on the combined use of apatinib and etoposide in patients with platinum-resistant recurrent ovarian cancer.^8^ Notably, future phase 3 trials could consider adjustments to the oral etoposide dosage. Additionally, we found that the QoL, SDS, and SAS scores of the patients did not differ significantly between pre- and post-treatment, suggesting that the combination therapy proposed in our study could help eliminate the need for infusions and hospital stays and promote home and outpatient care. This is important, since it may enhance the medication adherence, QoL, and mental well-being of the patients during treatment.

Our study used a 3-agent treatment regimen that combines immunotherapy with PD-L1 monoclonal antibodies, anti-angiogenic TKIs, and chemotherapy. PD-1/PD-L1 inhibitors work by blocking the PD-1/PD-L1 signaling pathway, which helps regulate the antitumor functions of T lymphocytes.^20^^,^^21^ Anti-­angiogenic therapy functions through targeting vascular endothelial growth factor (VEGF)/vascular endothelial growth factor receptor (VEGFR) signaling within the tumor microenvironment to induce vascular normalization pathways in the tumor microenvironment to normalize tumor blood vessels.^22^ Yang et al. previously identified a Jun proto-oncogene AP-1 transcription factor subunit (JUN/c-JUN)/VEGFR2 signaling axis, a direct interaction pathway between PD-L1 and VEGFR, that can impact anti-angiogenesis, suppress cell migration, and inhibit invasion.^23^ Additionally, specific DNA-damaging chemotherapies activate signaling pathways governing tumor cell survival, apoptosis, and innate immunity initiation, enhancing the antitumor immune response.^24^ The presence of living chemotherapy-damaged cells is crucial in T cell immunity, supporting the combination of chemotherapy with immunotherapy to promote effective T cell activation and anticancer immunity. This 3-agent combination disrupts the tumor’s immune tolerance, modifies the tumor microenvironment to facilitate immune cell infiltration and activity, and suppresses tumor cell metastasis and evasion, enhancing the effectiveness of ICIs.

### Strengths and limitations

The main strength of this study lies in its home-administered therapeutic protocol, which integrates subcutaneous envafolimab injections with oral lenvatinib and etoposide regimens. This tri-modality approach establishes a novel outpatient treatment paradigm for ovarian cancer management, demonstrating enhanced treatment feasibility while maintaining therapeutic intensity. However, our study also has several limitations. First, it was a single-arm trial without a control group, possibly resulting in potential selection biases in the study cohort. Second, the follow-up period was relatively short, and the sample size was small. Future studies with larger sample sizes and longer follow-up periods are warranted to validate our findings.

## Conclusion

Our study indicates that a 3-agent combination therapy with envafolimab, lenvatinib, and etoposide shows favorable therapeutic effects and manageable toxicity in patients with platinum-resistant recurrent ovarian cancer and represents a potential treatment option for at-home care.

## Supplementary Material

oyaf210_Supplementary_Data

## Data Availability

Requests for access to the clinical study data can be submitted via email to shenyang@seu.edu.cn.
