# Supplementary material for: Combination therapy for platinum-resistant ovarian cancer: a novel at-home regimen with envafolimab, lenvatinib, and etoposide
Source: Oncologist. 2025 Jul 14;30(9):oyaf210. doi: 10.1093/oncolo/oyaf210 (PMC12404298; doi:10.1093/oncolo/oyaf210)
Supplement: oyaf210_Supplementary_Data [file oyaf210_supplementary_data.zip › Supplementary_Tables_oncologist.docx]

| **Supplementary Table 1. Baseline characteristics of the patients, n(%)** | |
| --- | --- |
| Characteristics | Patients (n=18) |
| Age, years, median (range) | 54.5(41-75) |
| BMI, kg/m^2^, median (range) | 24.4(12-37) |
| ECOG performance status | |
| 0 | 5 (27.8) |
| 1 | 11 (61.1) |
| 2 | 2 (11.1) |
| Histology at diagnosis | |
| High-grade serous carcinoma | 15 (83.3) |
| Clear cell carcinoma | 3 (16.7) |
| FIGO stage at first diagnosis | |
| I | 2 (11.1) |
| II | 2 (11.1) |
| III | 10 (55.6) |
| IV | 4 (22.2) |
| Previous chemotherapy lines | |
| 1 | 6 (33.3) |
| 2 | 5 (27.8) |
| ≥3 | 7 (38.9) |
| Prior NACT | |
| Yes | 6(33.3) |
| No | 12 (62.5) |
| Prior bevacizumab | |
| Yes | 11 (61.1) |
| No | 7 (38.9) |
| Prior PARPIs | |
| Yes | 10 (55.6) |
| No | 8 (44.4) |
| Secondary surgery | |
| Yes | 3 (16.7) |
| No | 15 (83.3) |
| Regimen of the last chemotherapy | |
| Platinum-based | 13(72.2) |
| Non-platinum | 5 (27.8) |
| Intervals between the last chemotherapy and disease progression | |
| <3 months | 14 (77.8) |
| ≥3 months and <6 months | 4 (22.2) |
| Platinum-free intervals from the last platinum-based chemotherapy | |
| <6 months | 15 (83.3) |
| ≥6 months | 3 (16.7) |
| PD-L1 combined positive scores | |
| <1 | 3 (16.7) |
| ≥1 | 5 (27.8) |
| <10 | 5 (27.8) |
| ≥10 | 3 (16.7) |
| unknown | 8 (44.4) |
| **Abbreviations:** BMI, body mass index; ECOG, Eastern Cooperative Oncology Group; FIGO, The International Federation of Gynecology and Obstetrics; PARPIs, poly ADP ribose polymerase inhibitors; NACT, neoadjuvant chemotherapy. | |

| **Supplementary Table 2. Previous testing, therapies, and survival outcomes of the 18 patients** | | | | | | | | | |
| --- | --- | --- | --- | --- | --- | --- | --- | --- | --- |
| Patient No. | Histology | FIGO stage | Clinical response | PFS, months | HRD, score | PD-L1 CPS | BRCA1 | BRCA2 | Previous therapies |
| 01 | High-grade serous carcinoma | III | SD | 25 | NA | NA | NA | NA | DOC, DDP/CBP/GEM, OXA/paclitaxel liposome, CBP, niraparib/CPT-11, DDP/apatinib, VP-16/BEV, DOC/PEM, IFO |
| 02 | Clear cell carcinoma | II | PR | 9 | Negative | <1 | Negative | Negative | ADM, CBP |
| 03 | High-grade serous carcinoma | III | SD | 5.6 | NA | NA | Negative | Negative | Nab-p, apatinib/niraparib/OXA, BEV, anlotinib |
| 04 | High-grade serous carcinoma | III | SD | 1.8 | NA | NA | NA | NA | Paclitaxel liposome, OXA/apatinib/paclitaxel liposome, CBP/Nab-p, CBP, BEV/niraparib |
| 08 | Clear cell carcinoma | III | SD | 10.2 | Negative | 10 | Negative | Negative | Paclitaxel liposome, CBP |
| 09 | High-grade serous carcinoma | III | PD | 3 | NA | 0 | NA | NA | Paclitaxel liposome, CBP/anlotinib |
| 11 | High-grade serous carcinoma | III | CR | 24.6 | Positive | <1 | Negative | Positive | Paclitaxel liposome, CBP |
| 12 | Clear cell carcinoma | I | PR | 16 | NA | 10 | NA | NA | Paclitaxel liposome, CBP/DOC, LBP |
| 13 | High-grade serous carcinoma | II | SD | 16.2 | NA | NA | NA | NA | Paclitaxel liposome, CBP/olaparib/Nab-p, Bev |
| 14 | High-grade serous carcinoma | IV | PD | 2.6 | NA | 8 | NA | NA | TC |
| 15 | High-grade serous carcinoma | III | PR | 3.1 | Negative | 5 | Negative | Negative | Paclitaxel liposome, CBP, and niraparib |
| 18 | High-grade serous carcinoma | III | SD | 4.3 | NA | 10 | NA | NA | Paclitaxel liposome+CBP |
| 20 | High-grade serous carcinoma | IV | PD | 2.7 | NA | NA | Negative | Negative | Nab-p+CBP/ niraparib/ Paclitaxel liposome, DDP, BEV/VP-16 |
| 22 | High-grade serous carcinoma | I | PR | 9.1 | NA | NA | NA | NA | TC, anlotinib,/DOC, nedaplatin, BEV/Fluzoparib/ Nab-p, BEV/ Nab-p, BEV, LBP/ Nab-p, DDP |
| 24 | High-grade serous carcinoma | IV | SD | 1.7 | Positive | NA | Negative | Negative | ADM, CBP/BEV/Olaparib/ Nab-p, BEV,CBP/ADM, LBP/ IFO, Nab-p, BEV |
| 26 | High-grade serous carcinoma | IV | SD | 4.4 | NA | NA | Positive | Negative | TC/Olaparib, BEV/DOC, BEV, CBP |
| 27 | High-grade serous carcinoma | III | PR | 3.6 | NA | NA | NA | NA | Paclitaxel liposome, DDP, BEV/ GEM, BEV |
| 28 | High-grade serous carcinoma | III | PR | 3.0 | NA | NA | NA | NA | TC/ ADM, BEV |
| Abbreviations: FIGO, The International Federation of Gynecology and Obstetrics; PFS, progression-free survival; HRD, homologous recombination deficiency; CPS, combined positive score; SD, stable disease; PR, partial response; PD, progressive disease; DOC, docetaxel; DDP, cisplatin; CBP, carboplatin; GEM, gemcitabine; OXA, oxaliplatin; CPT-11, irinotecan; VP-16, etoposide; BEV, bevacizumab; PEM, pemetrexed; IFO, ifosfamide; ADM, doxorubicin; Nab-p, albumin-bound paclitaxel; LBP, lobaplatin; PTX, paclitaxel; TC, Ptx + Cbp; NA, not available. | | | | | | | | | |

| **Supplementary Table 3. Variations in psychological status and QoL of patients before and after 2-cycle treatment** | | | | |
| --- | --- | --- | --- | --- |
| Patient No. | Clinical response | SDS (*p* > 0.05) | SAS (*p*＞0.05) | FACT-O (*p*＞0.05) |
| 1 | SD | 52/45 | 12/11 | 113/120 |
| 2 | PR | 54/52 | 14/12 | 120/132 |
| 3 | SD | 60/62 | 17/18 | 118/123 |
| 4 | SD | 67/64 | 28/27 | 100/96 |
| 8 | SD | 69/63 | 23/22 | 103/98 |
| 9 | PD | 57/54 | 20/21 | 107/105 |
| 11 | CR | 42/42 | 16/13 | 122/122 |
| 12 | PR | 46/47 | 17/18 | 116/117 |
| 13 | SD | 51/53 | 21/18 | 102/100 |
| 14 | PD | 62/64 | 28/30 | 97/95 |
| 15 | PR | 56/53 | 21/22 | 117/120 |
| 18 | SD | 58/63 | 18/21 | 116/110 |
| 20 | PD | 52/48 | 16/16 | 102/108 |
| 22 | PR | 68/65 | 15/14 | 110/105 |
| 24 | SD | 53/55 | 13/12 | 108/106 |
| 26 | SD | 52/51 | 14/15 | 105/109 |
| 27 | PR | 51/49 | 16/14 | 120/122 |
| 28 | PR | 57/54 | 19/19 | 110/112 |
| **Abbreviations:** SDS, self-rating depression scale; SAS, self-rating anxiety scale; FACT-O, functional assessment of cancer therapy–ovarian cancer. Patients were assessed when they signed the informed consent form upon entering the group and during outpatient follow-up visits after two treatment cycles (42 days). | | | | |
