# Supplementary figures and images for: Combination therapy for platinum-resistant ovarian cancer: a novel at-home regimen with envafolimab, lenvatinib, and etoposide
Source: Oncologist. 2025 Jul 14;30(9):oyaf210. doi: 10.1093/oncolo/oyaf210 (PMC12404298; doi:10.1093/oncolo/oyaf210)

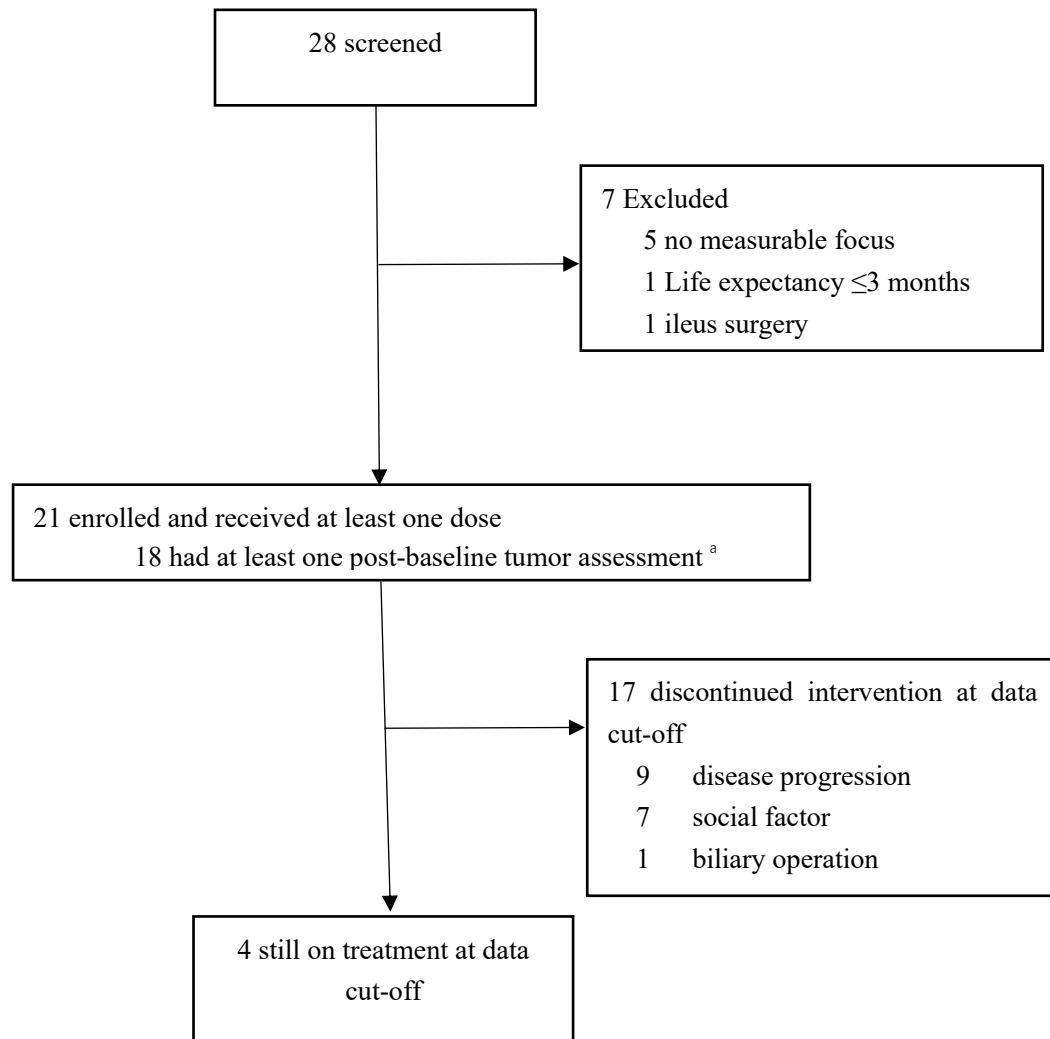

Supplement: oyaf210_Supplementary_Data [file oyaf210_supplementary_data.zip › Supplementary_figure_1_oncologist.pdf]
